# Functional cooperation between ASK1 and p21^Waf1/Cip1^ in the balance of cell-cycle arrest, cell death and tumorigenesis of stressed keratinocytes

**DOI:** 10.1038/s41420-021-00459-3

**Published:** 2021-04-12

**Authors:** Carlo De Blasio, Nagendra Verma, Marta Moretti, Samantha Cialfi, Azzurra Zonfrilli, Matteo Franchitto, Federica Truglio, Enrico De Smaele, Hidenori Ichijo, Isao Naguro, Isabella Screpanti, Claudio Talora

**Affiliations:** 1 Department of Molecular Medicine, Sapienza University of Rome, Viale Regina Elena 291, Rome, 00161 Italy; 2 Department of Experimental Medicine, Sapienza University of Rome, Viale Regina Elena 324, Rome, 00161 Italy; 3 Laboratory of Cell Signaling, Graduate School of Pharmaceutical Sciences, The University of Tokyo, Tokyo, Japan; 4 Present Address: IRCM, Institut de Recherche en Cancérologie de Montpellier, INSERM U1194, Université de Montpellier, Institut régional du Cancer de Montpellier, Montpellier, France

**Keywords:** Cell biology, Cancer

## Abstract

Both CDKN1A (p21 ^Waf1/Cip1^) and Apoptosis signal-regulating kinase 1 (ASK1) play important roles in tumorigenesis. The role of p21 ^Waf1/Cip1^ in attenuating ASK1-induced apoptosis by various stress conditions is well established. However, how ASK1 and p21 ^Waf1/Cip1^ functionally interact during tumorigenesis is still unclear. To address this aspect, we crossed *ASK1* knockout (ASK1KO) mice with *p21* ^Waf1/Cip1^ knockout (p21KO) mice to compare single and double-mutant mice. We observed that deletion of *p21* ^Waf1/Cip1^ leads to increased keratinocyte proliferation but also increased cell death. This is mechanistically linked to the ASK1 axis-induced apoptosis, including p38 and PARP. Indeed, deletion of *ASK1* does not alter the proliferation but decreases the apoptosis of p21KO keratinocytes. To analyze as this interaction might affect skin carcinogenesis, we investigated the response of ASK1KO and p21KO mice to DMBA/TPA-induced tumorigenesis. Here we show that while endogenous ASK1 is dispensable for skin homeostasis, ASK1KO mice are resistant to DMBA/TPA-induced tumorigenesis. However, we found that epidermis lacking both p21 and ASK1 reacquires increased sensitivity to DMBA/TPA-induced tumorigenesis. We demonstrate that apoptosis and cell-cycle progression in p21KO keratinocytes are uncoupled in the absence of ASK1. These data support the model that a critical event ensuring the balance between cell death, cell-cycle arrest, and successful divisions in keratinocytes during stress conditions is the p21-dependent ASK1 inactivation.

## Introduction

Non-melanoma skin cancer (NMSC) is considered the most common form of cancer with an increasing incidence world-wide^1^. However, the general incidence is not clear because various factors, including ultraviolet radiation exposure, behavior, and skin type might increase the risk of NMSC development^1^. Thus, the rising incidence associated with remarkable morbidity has generated great attention for unraveling the molecular pathways involved in NMSC cancer (^1^ and references therein). The genetic lesions that characterize skin carcinogenesis are still not fully understood. However, while basal cell carcinomas (BCCs) are believed to develop de novo, skin squamous cell carcinomas (SCCs) development is viewed as a multistep process^2^. In line with this view, multiple and interacting environmental agents have been shown to play a potential role in skin cancer development^2^. Nonetheless, evidence for a specific signal transduction pathway forcing SCC development is still missing. Apoptosis signal-regulating kinase 1 (ASK1) was identified as a serine/threonine kinase that activates the c-Jun N-terminal kinase (JNK)- and p38-associated signaling pathways^3–5^. ASK1 activation occurs in a wide range of cellular responses, including apoptosis, cell differentiation, as well as cell survival and production of inflammatory cytokines^5^. Thus, it is not surprising that ASK1 deregulation has been linked to the development of several human diseases, including cancer^5^. p21 regulates multiple cellular functions that can have opposite effects from ASK1 and protects cells from genotoxic and other cellular stresses. In skin cancer, it has been reported that ASK1 has an important role in promoting skin tumorigenesis^6,7^. ASK1 acts as a tumor promoter factor by stimulating the production of inflammatory cytokines, such as TNF-*α*, IL-6^6^. It was found that the association of p21 with ASK1 could attenuate rapamycin-induced cellular stress in both human and murine cells^8^. Although the mechanism by which p21 determines ASK1 inhibition is well understood, the effect of this functional interaction as well the downstream effects in tumorigenesis particularly in skin cancer is largely unknown. Here, investigating the roles of ASK1 and p21 in chemically induced skin tumorigenesis in mice, we suggest that ASK1 and p21 are critically involved in skin carcinogenesis by differentially regulating keratinocyte apoptosis and proliferation.

## Results

### Inactivation of both p21 and ASK1 has not a major impact on differentiation of keratinocytes

**Fig. 1:**
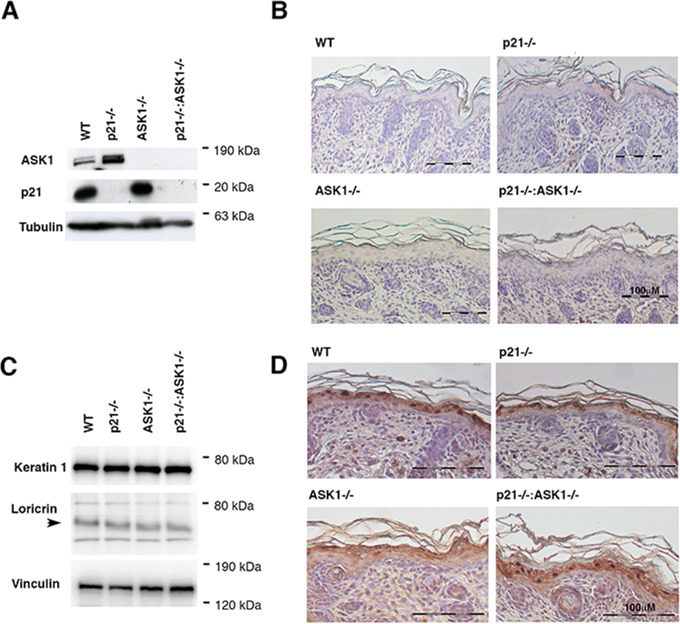
Inactivation of both *p21* and *ASK1* has not a major impact on differentiation of keratinocytes. **A** Primary mouse keratinocytes were established from 2-day-old newborn mice and maintained in low calcium medium. Cells were analyzed for western blot analysis with the indicated antibodies 5 days post-seeding. **B** H&Es taining of single p21KO, ASK1KO, ASK1/p21KO, and wild-type (WT) skin at birth (2-day-old newborn mice). 10X microscope field (scale bar: 100 μm). **C** Equal amounts of protein extract from WT, p21KO, ASK1KO ASK1/p21KO neonate dorsal skin (2-day-old newborn mice) were separated by SDS-PAGE and analyzed by western blot with antibodies directed against keratin 1 and loricrin. Vinculin has been used as a loading control (**D**) Immunohistochemical labeling of dorsal skin sections from 2-day-old newborn WT, p21KO, ASK1KO ASK1/p21KO mice using antibodies specific to loricrin shows a similar expression level and localization of the differentiation marker in WT and KO mice. 20X microscope field (scale bar: 100 μm).

To generate ASK1/p21 double KO mice, we crossed ASK1KO mice with p21KO mice. We were able to produce mice with both *p21* and *ASK1* null alleles (ASK1/p21KO); breeding produced offspring at the expected Mendelian frequency, mice were viable fertile and exhibited no apparent abnormalities in the tissues and organs examined including the skin. To verify the absence of *p21* and *ASK1* gene expression in the ASK1/p21KO mice we performed western blot analyses in primary keratinocytes derived from WT, p21KO, ASK1KO, and ASK1/p21KO. As shown in Fig. 1A, western blot analysis using antibodies raised against the ASK1 and p21 proteins did not reveal their expression in the ASK1/p21KO primary keratinocytes. To analyze potential morphological changes in the skin, biopsies derived from newborn ASK1/p21KO and control genotypes mice were harvested for histological analysis. As shown in Fig. 1B, no structural changes could be detected in the skin of ASK1/p21KO mice. Specifically, the morphology of the dermis did not significantly differ. Finally, no epidermal hyperplasia or atypical keratinocytes were identified. Therefore, no gross abnormalities were observed in the dermis or the epidermis. Observation of ASK1/p21KO mice for a long time period (a year) did not reveal any spontaneous skin phenotype (data not shown). To further investigate the effect of the double ASK1/p21 null mutation on epidermal differentiation, we examined the expression of differentiation markers by western blot and immunochemistry (Fig. 1C and D). No significant difference in the expression of early (keratin 1) and late (loricrin) differentiation markers were observed in skin from WT, p21KO, ASK1KO, and ASK1/p21KO 2-day old newborn mice by western blot analysis (Fig. 1C). In agreement with this observation, loricrin was similarly expressed in WT, p21KO, ASK1KO, and ASK1/p21KO (Fig. 1D). These results indicate that genetic inactivation of both *p21* and *ASK1* has not a major impact on differentiation of keratinocytes.

### Increased proliferation and reduced apoptosis of ASK1/p21KO primary keratinocytes

**Fig. 2:**
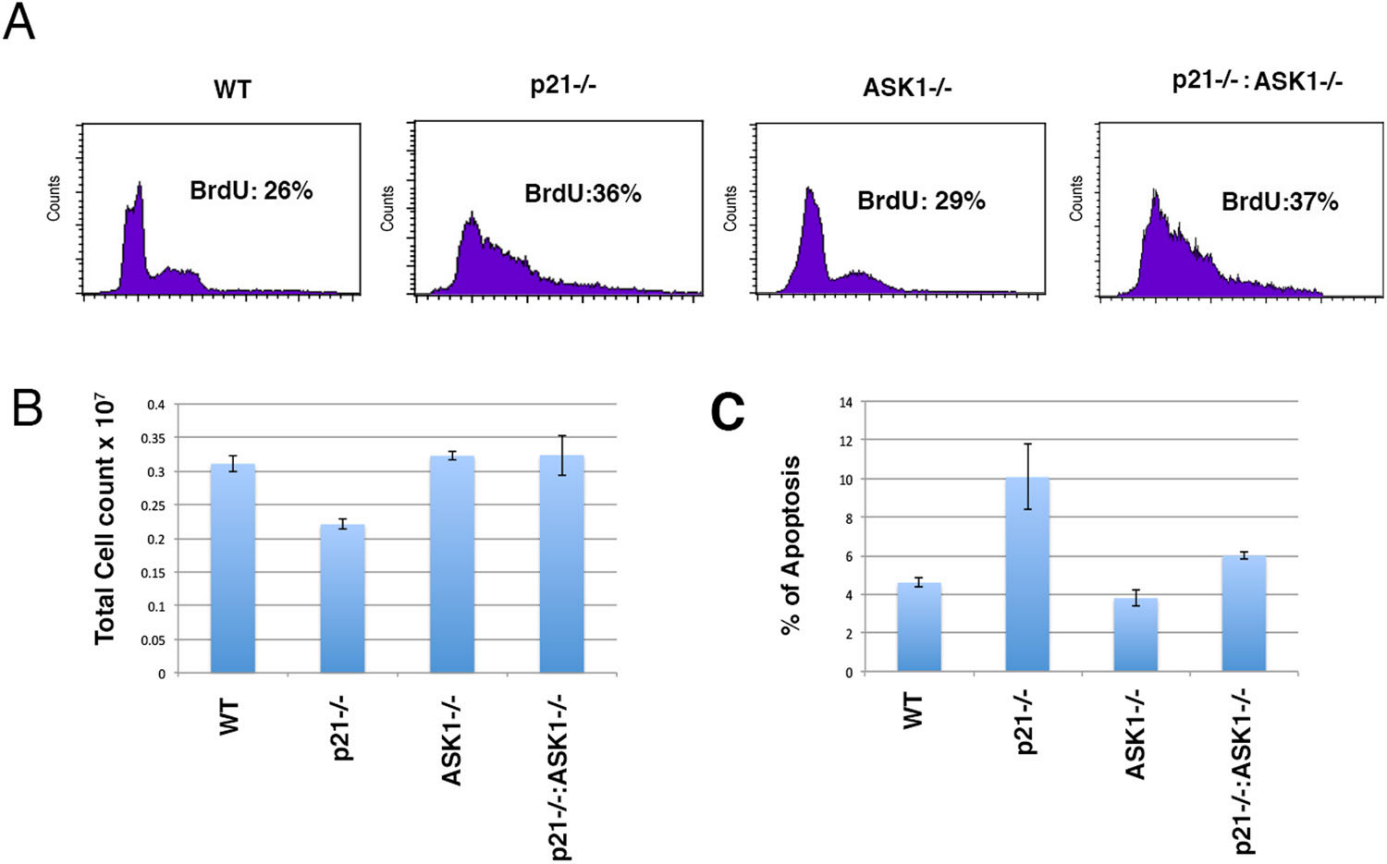
Increased apoptosis but not increased proliferation of p21-deleted primary keratinocytes is ASK1-dependent. **A** Primary mouse keratinocytes were established from 2-day-old newborn mice and maintained in low calcium medium. Cells were analyzed 5 days post-seeding. Cultured keratinocytes cells were pulse labeled with BrdU for 12 h, harvested and prepared for FACS analysis as described in Materials and methods. **B** Primary mouse keratinocytes were established from 2-day-old newborn mice and maintained in low calcium medium. Cells were seeded in triplicate at a density of 4 × 10^4^ cells/cm^2^ in 6-well culture dishes and analyzed 10 days post-seeding. Once the cells were trypsinized approximately 0.2 mL of the cells were mixed with an equal volume of 0.4% Trypan blue solution and viable cells were counted using a hemocytometer. Results are expressed as mean ± SEM of triplicate experiments. **C** Flow cytometric analysis of the cell death phenotype by PI staining, the % of apoptotic cells is indicated. Results are expressed as mean ± SEM of triplicate experiments.

To further characterize how the concomitant deletion of *ASK1* and *p21* might affect keratinocyte behavior WT, ASK1KO, p21KO, and ASK1/p21KO primary keratinocytes were analyzed. Asynchronous keratinocyte cultures in low calcium medium (0.03 mM) were either pulse labeled with BrdU for 12 h or propidium iodide stained. Flow cytometry analysis was then performed to investigate the proliferation and apoptosis of WT, ASK1KO, p21KO, and ASK1/p21KO. The results (Fig. 2A) demonstrate an increased labeling index for both p21KO and ASK1/p21KO keratinocytes when compared with the WT and ASK1KO keratinocytes. p21 acts as both positive and negative regulators of apoptosis^9^. Interestingly, p21KO keratinocytes number declined faster than WT, ASK1KO, and p21ASK1KO (Fig. 2B), suggesting an inhibiting role of p21 in apoptosis. To further confirm this observation, similar numbers of keratinocytes of each genotype were plated and cultured in low calcium medium, and the number of viable and apoptotic cells was determined at 4 day after plating. An increased apoptotic rate in p21KO keratinocytes was observed (Fig. 2C). Interestingly, both ASK1KO and ASK1/p21KO keratinocytes showed a percentage of apoptotic cells similar to WT keratinocytes (Fig. 2C). These results demonstrate that the lack of p21 leads to increased keratinocyte proliferation and apoptosis.

### p21 maintains keratinocytes cell viability by restraining ASK1/p38 proapototic signaling

**Fig. 3:**
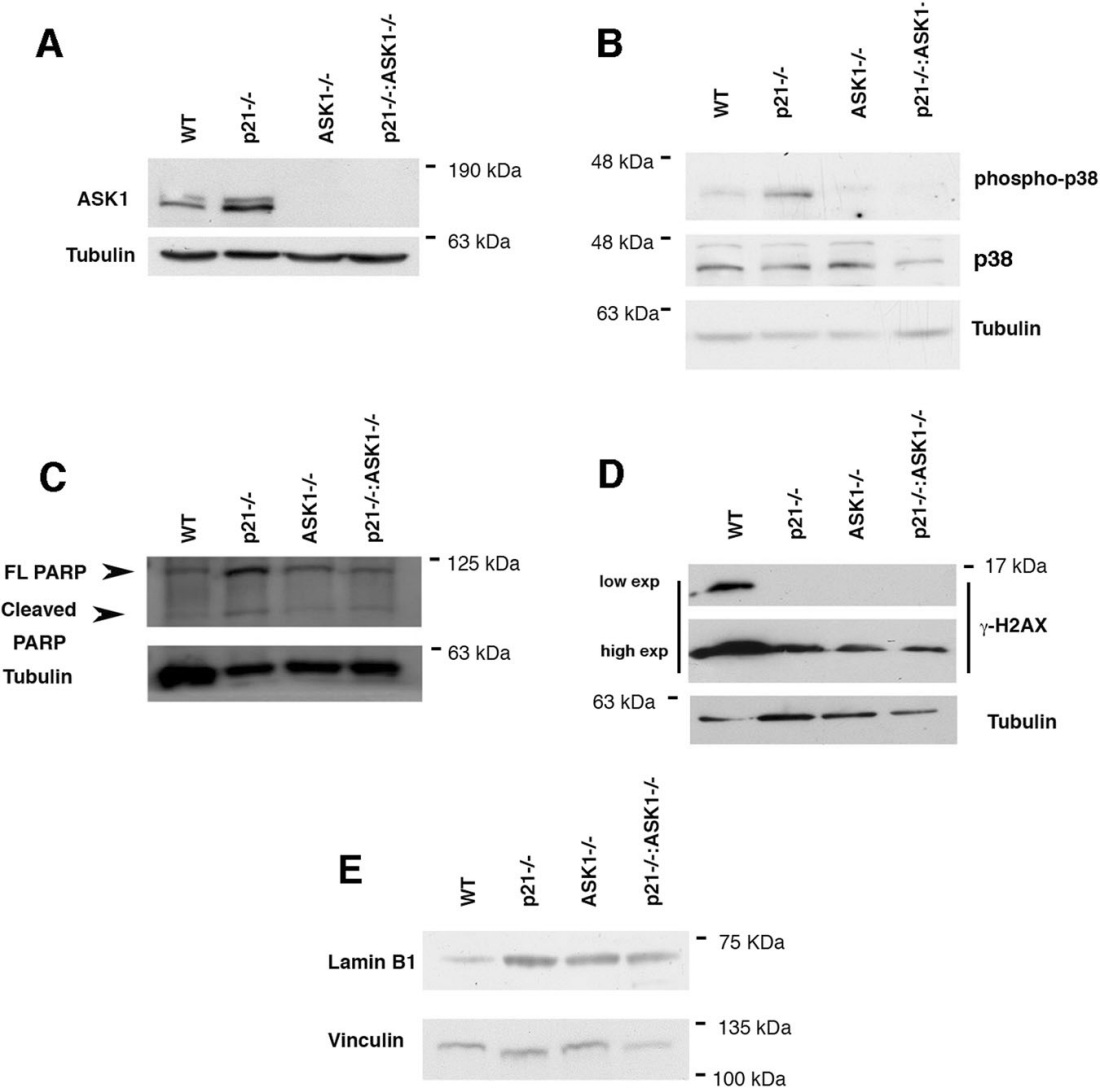
ASK1-p38 axis promotes apoptosis in p21-deficient primary keratinocytes. **A**–**E** Primary mouse keratinocytes were established from 2-day-old newborn mice and maintained in low calcium medium. Cells were analyzed 5 days post-seeding by western blot analysis with the indicated antibodies.

Then we set out to understand the mechanism of cell death induction in p21KO keratinocytes. We hypothesized that an increase in the level of the pro-apoptotic activity of ASK1 in the p21KO keratinocytes might account for this effect. Therefore we assessed the expression levels of ASK1 as well as proteins involved in apoptotic response whose activity has been shown to be ASK1-dependent: p38 and its downstream targets Caspase-3 and PARP^3^. Immunoblotting revealed a substantial increase in the expression level of ASK1 in p21KO keratinocytes (Fig. 3A). ASK1 increase was accompanied by an increase in the expression levels of phosphorylated p38 (p-p38) (Fig. 3B). To further assess the apoptotic nature of cell death in p21KO keratinocytes, we evaluated the expression of the apoptotic effector, PARP. We found that the levels of this apoptotic marker were increased in p21KO keratinocytes (Fig. 3C). Interestingly, p21/ASK1KO keratinocytes showed a decreased level in both p-p38 and cleaved PARP expression (Fig. 3B, C), indicating that an ASK1/p38/PARP dependent apoptotic pathway was activated in response to the loss of p21 expression. As cellular senescence is associated with both increased p21 level and activation of the ASK1/p38 axis^10–12^ we aimed to examine whether p21/ASK1 deficiency was paralleled by blunted senescence. Although there is not a universal biomarker that might be used unequivocally to quantify cellular senescence both increased γ-H2AX (the phosphorylated form at the C-terminal serine-139 of the H2AX histone) and Lamin B1 loss have been associated with cellular senescence^13–16^. Thus we analyzed the expression of these markers in p21KO, ASK1KO, and p21/ASK1KO derived keratinocytes relatively to the WT keratinocytes. In line with a role of both p21 and ASK1 in senescence, we observed loss of *γ*-H2AX expression and increased Lamin B1 expression in p21KO, ASK1KO, and p21/ASK1KO keratinocytes (Fig. 3D–E). Overall, our findings indicate that deficiency of ASK1 as well as p21 results in blunted senescence in KCs. Therefore it appears that the balance of ASK1/p21 activity in keratinocytes promotes the balance between senescence and apoptosis.

### ASK-1 functions as a tumor suppressor by facilitating DMBA-induced apoptosis in epidermal keratinocytes

**Fig. 4:**
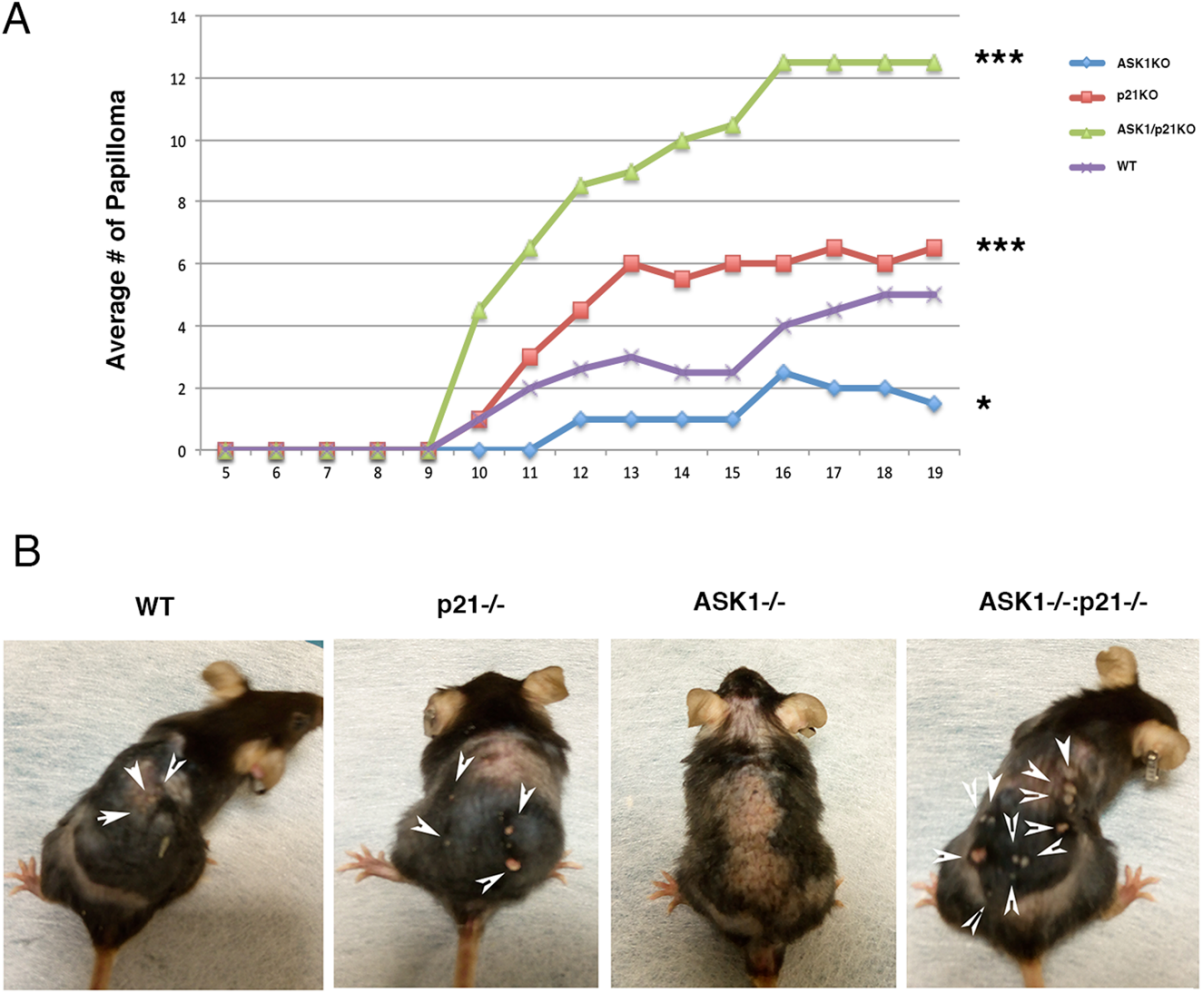
Inactivation of *both p21 and ASK1* has a major impact on DMBA/TPA-induced carcinogenesis. **A** In vivo DMBA/TPA induces skin carcinogenesis in 7–9 week old mice. Upper panel the indicated mice were treated once with DMBA (100 μg in 200 μl of acetone) and then continually treated with TPA (10 μg in 200 μl of acetone) twice a week for 20 weeks. The average (8 mice each group) number of papillomas per mouse is shown. The significance of the differences was calculated using one-way ANOVA. *P < 0.05, ***P < 0.0005 are the significance of indicated mice compared with WT mice. **B** Lower panel, a representative picture of the treated mice.

A series of studies have indicated that ASK1 plays an important promoting role in many stress-related diseases. Conversely, p21 exerts a protective function against stress. The ASK1 and p21 functions have opposite effects on both cellular stress responses and skin cancer. ASK1 promotes skin cancer while p21 functions as a suppressor of malignant skin tumor formation. Alterations in any of these pathways in stressed cells can shift the balance in favor of survival or death. The DMBA/TPA*-*induced carcinogenic process in the skin is linked mainly to the disruption of the physiological balance in pro- and anti-stressor signaling pathway^17^. Thus, we wished to examine the contribution of ASK1 and p21 in skin carcinogenesis induced by DMBA/TPA. Tumors were initiated with a single dose of DMBA and promoted with TPA twice per week^17^. The average number of tumors was considerably lower in ASK1KO than in both WT mice and p21KO (Fig. 4A, B). As the deletion of *p21* in the epidermis increases tumorigenesis^9,18,19^, and p21 forms an inhibitory complex with ASK1^20^, we wondered whether the complex is essential for the effects described above. Therefore, we investigated whether there is a genetic interaction between *ASK1* and *p21* to DMBA/TPA-induced tumorigenesis. The average number of tumors was considerably higher in p21KO than in wild-type mice; we detected an additional effect of *p21* deletion in the absence of *ASK1* with an increased average number of tumors compared with p21KO mice indicating a genetic interaction under these circumstances.

## Discussion

The genetic lesions that characterize skin carcinogenesis are still not fully understood. Cell stress response represents an integral part of the cellular physiology to either ensure the cell’s survival or alternatively eliminate damaged cells. Hence, alteration of the mechanisms that support cellular stress response is tightly linked to many common human diseases, including cancer. Thus, identification of the molecular mechanisms that a cell mounts to support survival or death as a protective responses to counteract the effect of the stress on cellular processes and how the disturbance of this balance may result in either protumor or antitumor outcome is of interest in cancer biology. Herein, we provide evidence that contributes to support a role of the p21/ASK1 axis to tumor suppressor activities and paradoxical tumor-promoting activities, and how the disturbance of their functional interaction is implicated in stress-induced cancer in keratinocytes. Herein, we found that, no gross abnormalities were observed in the epidermis of ASK1/p21 double KO mice. In contrast, we found several abnormalities in growth/apoptotic properties of ASK1/p21 double KO keratinocytes indicative of their malignant potential. It is known that p21 promotes cell-cycle arrest in response to many stimuli^9,21^. However, p21 also inhibits apoptosis, which might account for its paradoxical oncogenic activities^9,21^. In particular, p21 binds proteins directly involved in the induction of apoptosis, including the proapototic ASK1/p38 axis inhibiting their activity^9,20^. Interestingly, we found that *p21* deletion increased the level of BrdU labeling in cultured primary keratinocytes, but also increased the levels of the ASK1/p38 axis and cleaved PARP1, supporting the important role of p21 in both promoting cell-cycle control and protecting keratinocytes from apoptosis. An active proliferation state exposes cells to the problem of an elevated stress condition (e.g. oxidative stress) associated with meeting the need of energy demand^22^. ASK1 is activated under various stress conditions including oxidative stress^23^. Interestingly, we found that *ASK1* deletion reduced the apoptosis observed in p21KO keratinocytes, but in contrast ASK1/p21 double KO-derived keratinocytes continued to incorporate BrdU at a high level as observed in p21KO keratinocytes. These findings indicate that ASK1 is an important mediator of p21-deficiency-induced apoptosis and also indicate that the apoptotic and proliferative phenotype of p21KO keratinocytes can be uncoupled.

Accumulating evidence has shown that both p21 and ASK1 mediate cellular senescence^11,12^. Aberrant DNA proliferation and DNA damage accumulation are involved in senescence induction and play a crucial role in restricting the proliferation of damaged precancerous cells^24,25^. Thus, cellular senescence represents a tumor-suppressive mechanism that counteracts the risk for malignant transformation^26^. Herein, we observed that double p21/ASK1 deletion might blunt a senescence-like phenotype, since p21/ASK1KO derived keratinocytes displayed loss of *γ*H2AX expression and increased Lamin B1 expression. Furthermore, we also observed that ASK1/p21KO derived keratinocytes displayed an increased level of BrdU labeling and reduced apoptosis, suggesting an important role of p21/ASK1 signaling in the senescence program that locks the keratinocytes into a cell-cycle arrest to prevent malignant transformation. Accordingly, we found that loss of both p21 and ASK1 increased the rate of DMBA/TPA-induced skin tumorigenesis. Our observations support a role of the p21/ASK1 axis in senescence of keratinocytes that might be involved in preventing the risk of tumorigenesis.

**Fig. 5:**
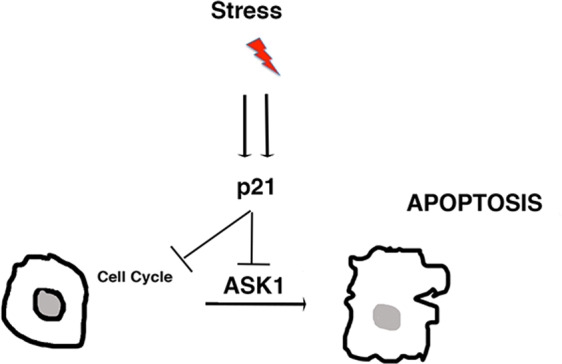
Model. When exposed to environmental stress or aging cultured primary mouse keratinocytes undergo a growth arrest triggered by p21. However, under stress conditions p21 counteracts ASK1-dependent apoptosis. In contrast to wild-type keratinocytes, when exposed to stress p21-deficiency impedes the block of DNA synthesis. Thus, the most severely affected p21-deficient cells are eliminated in the course of stress by ASK1-activation. We propose that ASK1 and p21 function as a checkpoint-tumor surveillance mechanism in which ASK1 represents a mechanism for compensating loss of p21 activity. Thus, loss of the p21/ASK1 axis impairs tumor surveillance with increasing the risk of tumorigenesis.

In conclusion, our study supports the hypothesis that primary mouse keratinocytes rely on both ASK1 and p21 for the induction of senescence. Additionally, we hypothesize that the p21 deficiency strongly impedes both the induction of senescence and cell-cycle arrest. However, if stress persists p21-deficient cells in the population are eliminated by ASK1/p38- dependent apoptosis (Fig. 5). Together these data indicate the deregulation of the p21/ASK1 axis in skin stress-initiated tumorigenesis.

## Materials and methods

### Animals

ASK1 deficient (ASK1 KO) p21 deficient (p21 KO) mice and C57BL/6 N wild-type mice were used in the present study. Generation of mice deficient for the *ASK1 (MAP3K5)* gene has been previously described^23^. p21 KO mice reference stock # 016565 were purchased from the Jackson Labortory, (Bar Harbor, ME USA). All animal protocols were approved by the Institutional Animal Care authorities-Department of Molecular Medicine Sapienza—University of Rome and conducted in accordance with Italian Governing Law (D.lgs 26/2014; Prot. no. 03/2013). Autorizzazione del Ministero della Salute n. 552-2016-PR prot. C1368.0 del 05/09/2019.

### Primary mouse keratinocytes

Primary mouse keratinocytes were established from 2-day-old newborn mice using the CELLNTEC reagents according to the manufacturer’s instructions (CELLNTEC, Bern, Switzerland). Keratinocytes were maintained in primary mouse keratinocytes CnT-07 medium (CELLNTEC, Bern, Switzerland). Cells were analyzed 5 days post-seeding.

### Flow cytometry analysis

Keratinocytes (1 × 10^6^) were washed in 1% BSA, fixed in chilled 70% ethanol, treated with RNase A (100 μg/ml RNase) for 15 min at 37 °C harvested and resuspended in cell staining buffer (Cat# 420201) and 25 µl of Propidium Iodide Solution (Cat# 421301) in the dark for 30 min at room temperature according to the manufactory instruction of Propidium Iodide Solution staining KIT (Biolegend San Diego, CA, USA). Data acquisition and analysis were carried out using a flow cytometry. Data analysis was performed with CellQuestPro software (BD Biosciences, Milan, Italy). For 5-bromo-2′-deoxyuridine (BrdU) proliferation assay, primary keratinocytes were established from 2-day-old newborn mice using the CELLNTEC reagents according to the manufacturer’s instructions (CELLNTEC, Bern, Switzerland). Keratinocytes were maintained in primary mouse keratinocytes CnT-07 medium (CELLNTEC, Bern, Switzerland). Cells were analyzed 5 days post-seeding by loading cells with 5 μg/ml of BrdU. Cells were incubated for further 12 h and analyzed according to the manufacturer instructions of 5-bromo-2′-deoxyuridine -BrdU proliferation KIT (Biolegend San Diego, CA, USA).

### Reagents and immunoblotting

The following reagents were purchased from Santa Cruz Biotechnology, Santa Cruz, CA, U.S.A: vinculin, tubulin, p21Waf1/Cip. ASK1 antibodies were purchased from Abcam (Cambridge, MA, U.S.A.) Loricirn and Keratin 1 were purchased from (Biolegend San Diego, CA, USA) and PARP, phospho-p38, p38, Lamin B1, and yH2AX were purchased from Cell Signaling Technology (Beverly, MA, U.S.A). All cell extracts were prepared according to the manufacturer’s instructions for detection of phosphor-ERK (Cell Signaling Technology, Beverly, MA, U.S.A.) as previously described^21,27^. Briefly, culture cells were lysed in Laemmli Buffer (Biorad, Hercules, CA, USA) immediately scraped on ice and cell extracts transferred to a microfuge tube; after sonication for 10–15 s to shear DNA, the sample was heated for 5 min at 95 °C and briefly centrifuged. Total extracts were analyzed by SDS-PAGE gel. For whole-skin protein extraction, skin section from 2-day-old newborn mice was frozen in liquid nitrogen and then crushed to powder using a mortar and pestle; pulverized skin was then lysed adding Laemmli Buffer (Biorad, Hercules, CA, USA) and further processed with the cell extract preparation method described above.

### Histology

For Hematoxylin and eosin (H&E) and immunostaining using paraffin-embedded tissue sections from 2-day-old newborn mice samples from various mutant and wild-type animals were fixed in 4% paraformaldehyde in PBS, dehydrated with ethanol and embedded in paraffin, which was then sectioned at 10 μm. Skin sections were processed for H&E staining or probed with specific Loricrin (clone Poly19051) antibodies (Biolegend San Diego, CA, USA), according to the manufactory instruction of Hematoxylin and Eosin staining KIT-HAE or staining HRP ready to use kit (MTM001, ScyTek Laboratories, Logan, UT, USA) respectively.

### Chemical skin carcinogenesis studies

For DMBA/TPA experiments from various mutant and wild-type animals background, mutant mice and their age-matched littermate controls were treated with standard protocols for skin chemical carcinogenesis models as previously described^17^.

### Statistical analysis

Statistical analysis between two groups was performed using a Student’s *t* test, between multiple groups using a one-way analysis of variance (ANOVA). For all analyses, *p* ≤ 0.05 was accepted for statistical significance.

## Supplementary information

Related Manuscript File
