# Supplementary material for: Functional cooperation between ASK1 and p21Waf1/Cip1 in the balance of cell-cycle arrest, cell death and tumorigenesis of stressed keratinocytes
Source: Cell Death Discov. 2021 Apr 12;7:75. doi: 10.1038/s41420-021-00459-3 (PMC8042117; doi:10.1038/s41420-021-00459-3)
Supplement: Supplementary file 1 — Related Manuscript File [file 41420_2021_459_MOESM1_ESM.pdf]

DECLARATION OF CONTRIBUTIONS TO ARTICLE

ADMC

Manuscript Number: CDDISCOVERY-21-1302R

Journal Name: *Cell Death Discovery* (the 'Journal')

Proposed Title of the Contribution:

Functional cooperation between ASK1 and p21Waf1/Cip1 in the balance of cell cycle arrest, cell death and tumorigenesis of stressed keratinocytes (the 'Contribution)

Author(s):

Carlo De Blasio, Nagendra Verma, Marta Moretti, Samantha Cialfi, Azzurra Zonfrilli, matteo franchitto, federica truglio, Enrico De Smaele, Isao Naguro, Hidenori Ichijo, Isabella Screpanti and Claudio Talora (the 'Authors')

For all *CDDis*covery articles, each person named as an author in the published version must be able to show he or she has contributed substantially to the article.

Authorship credit should be based on 1) substantial contributions to conception and design, acquisition of data, or analysis and interpretation of data; 2) drafting the article or revising it critically for important intellectual content; and 3) final approval of the version to be published. Authors should meet conditions 1, 2 and 3.

Any person who cannot be shown to have made a substantial contribution to the article cannot be listed as an author in the final version. The name of any person who is deemed to have made a minor contribution can, however, appear in the Acknowledgments section of the article.

Please complete the table below to indicate the contributions of all named authors to the manuscript.

Author Full Name:	Specification of Contribution to the Manuscript:
Carlo De Blasio	Performed experiments
Nagendra Verma	Performed experiments
Marta Moretti	Performed experiments
Samantha Cialfi	Performed experiments
Azzurra Zonfrilli	Performed experiments
Matteo Franchitto	Performed experiments
Federica Truglio	Performed experiments
Enrico De Smaele	Commented on the paper with critically important intellectual content and provided critical reagents
Isao Naguro	Provided ASK1 mice and provided critical feedback
Hidenori Ichijo	Provided ASK1 mice and provided critical feedback
Isabella Screpanti	Commented on the paper with critically important intellectual content and provided critical reagents
Claudio Talora	Designed the research, analyzed the data, assembled the figures and wrote the paper;

Please complete the table below to indicate the contributions of all named authors to the figures.

Figure 1:

Figure 1. Inactivation of both p21 and ASK1 has not a major impact on differentiation of keratinocytes.
A) Primary mouse keratinocytes were established from 2-day-old newborn mice and maintained in low calcium medium. Cells were analyzed for western blot analysis with the indicated antibodies 5 days post-seeding. B) H&E staining of single p21KO, ASK1KO, ASK1/p21KO and wild-type (WT) skin at birth (2-day-old newborn mice). 10X microscope field (scale bar: 100µm). C) Equal amounts of protein extract from WT, p21KO, ASK1KO ASK1/p21KO neonate dorsal skin (2-day-old newborn mice) were separated by SDS-PAGE and analyzed by western blot with antibodies directed against keratin 1 and loricrin. Vinculin has been used as a loading control (D) Immunohistochemical labeling of dorsal skin sections from 2-day-old newborn WT, p21KO, ASK1KO ASK1/p21KO mice using antibodies specific to loricrin shows a similar expression level and localization of the differentiation marker in WT and KO mice. 20X microscope field (scale bar: 100µm).

Carlo De Blasio and Claudio Talora have contributed to the generation of double KO mice. Samantha Cialfi and Azzurra Zonfrilli performed the expeirments in pane A and Panel C. Marta Moretti performed the experiments in Panel B and D.

Figure 2:

Figure 2. Increased apoptosis but not increased proliferation of p21-deleted primary keratinocytes is ASK1-dependent.
A) Primary mouse keratinocytes were established from 2-day-old newborn mice and maintained in low calcium medium. Cells were analyzed 5 days post-seeding. Cultured keratinocytes cells were pulse labeled with BrdU for 12 h, harvested and prepared for FACS analysis as described in Materials and methods. B) Primary mouse keratinocytes were established from 2-day-old newborn mice and maintained in low calcium medium. Cells were seeded in triplicate at a density of 4x104 cells/cm2 in 6-well culture dishes and analyzed 10 days post-seeding. Once the cells were trypsinized approximately 0.2 mL of the cells were mixed with an equal volume of 0.4% Trypan blue solution and viable cells were counted using a hemocytometer. Results are expressed as mean ± SEM of triplicate experiments. C) Flow cytometric analysis of the cell death phenotype by PI staining, the % of apoptotic cells is indicated. Results are expressed as mean ± SEM of triplicate experiments.

Nagendra Verma and Claudio Talora performed the experiments presented in panel A, B and C

Figure 3:

Figure 3. ASK1-p38 axis promotes apoptosis in p21-deficient primary keratinocytes.
A,B,C,D,E) Primary mouse keratinocytes were established from 2-day-old newborn mice and maintained in low calcium medium. Cells were analyzed 5 days post- seeding by western blot analysis with the indicated antibodies.

Azzurra Zonfrilli, Samantha Cialfi , Matteo Franchitto and Federica Truglio performed the experiments presented in this figures raging form culturing primary keratinocytes to western blot.

Figure 4:

Figure 4. Inactivation of both p21 and ASK1 has a major impact on DMBA/TPA induced carcinogenesis.
A) In vivo DMBA/TPA induces skin carcinogenesis in 7- 9 week old mice. Upper panel the indicated mice were treated once with DMBA (100 µg in 200 µl of acetone) and then continually treated with TPA (10 µg in 200 µl of acetone) twice a week for 20 weeks. The average (8 mice each group) number of papillomas per mouse is shown. The significance of the differences was calculated using one-way ANOVA.
* P< 0.05, *** P< 0.0005 are the significance of indicated mice compared with WT mice. B) Lower panel, a representative picture of the treated mice.

Claudio Talora, Carlo De Blasio and Nagendra Verma performed the DMBA/TPA induced carcinogensis experiments and perfomed statistical analysis.

Figure 5:

Figure 5. Model.
When exposed to environmental stress or aging cultured primary mouse keratinocytes undergo a growth arrest triggered by p21. However, under stress conditions p21 counteracts ASK1-dependent apoptosis. In contrast to wild-type keratinocytes, when exposed to stress p21-deficiency impedes the block of DNA synthesis. Thus, the most severely affected p21-deficient cells are eliminated in the course of stress by ASK1-activation. We propose that ASK1 and p21 function as a checkpoint-tumor surveillance mechanism in which ASK1 represents a mechanism for compensating loss of p21 activity. Thus, loss of p21/ASK1 axis impairs tumor surveillance with increasing the risk of tumorigenesis.

CLaudio Talora conceived the model.

Figure 6:

Signed for and on behalf of the Author(s):	Print Name:	Date:
[signature]	Claudio Talora	5 March 2021
